# Note-Taking Skill Among Bilingual Students in Academia: Literacy, Language and Cognitive Examination

**DOI:** 10.3389/fpsyg.2019.00870

**Published:** 2019-05-14

**Authors:** Mona Asaly-Zetawi, Orly Lipka

**Affiliations:** ^1^Department of Learning Disabilities, University of Haifa, Haifa, Israel; ^2^Edmond J. Safra Brain Research Center, Department of Learning Disabilities, University of Haifa, Haifa, Israel

**Keywords:** note-taking, post-secondary, bilingual students, literacy, lecture

## Abstract

The ability to take notes while listening to a lecture is important and complicated. The main goal of the current study was to examine note-taking skills among students with Hebrew as a first language (L1) and students with Arabic as a first language and Hebrew as a second language (L2). Literacy, language, cognitive, and note-taking skills were assessed among 63 undergraduate students (28 L1). L1 students were found to produce notes of higher quality than L2 students. Moreover, there were significant differences between the groups on measures of vocabulary, word reading fluency, and handwriting speed. The results also revealed that first language was the most important variable in predicting note quality, followed by word reading fluency. Educational implications and directions for further research are discussed in light of the findings.

## Introduction

Post-secondary education has become an increasingly important pre-requisite to full participation in today’s job market. One of the key factors enabling successful learning in the academic world is the ability to take notes during lectures (Dunkel and Davy, 1989; Carrell et al., 2004). Note-taking is one of the main ways that students acquire knowledge and a powerful way for them to gain control over their own learning (Burns and Sinfield, 2012). Furthermore, it is an active and multifaceted skill that requires academic listening and purposeful attendance to the speaker (Gur et al., 2013). The note-taking process requires students to write fast enough to keep up with the pace of the lecture, pay attention, decide what is important to record, and make sense of their notes after class (Suritsky, 1992). Studies indicate that the review of notes following a lecture results in better recall of lecture material (Howe, 1970) and that better note-taking contributes to higher test scores (Fisher and Harris, 1973; Rickards and Friedman, 1978; Kiewra and Fletcher, 1984; Bretzing et al., 1987; Kiewra et al., 1991; O’Donnell and Dansereau, 1993; Peverly et al., 2003, 2007; Titsworth and Kiewra, 2004). Furthermore, note-taking quality predicts academic performance among students (Kobayashi, 2006; Peverly et al., 2007; Peverly and Sumowski, 2012). Rahmani and Sadeghi (2011) have demonstrated the importance of training on how to take notes, which improves performance on comprehension and retention tests.

Note-taking is considered a crucial skill for students in middle and high school, and the primary resource for learning content at college and university (Buttrill et al., 1989). Unsurprisingly, most college students consider note-taking during lectures as an essential educational activity (Dunkel and Davy, 1989) and almost all of them take notes in class (Palmatier and Bennett, 1974). Taking notes is considered a strategy for increasing attention while listening to a lecture and for retaining its content (Dunkel and Davy, 1989). When students are preparing for high-stakes exams, they largely rely on memorizing their notes (Karpicke et al., 2009; Morehead et al., 2016). A recent study that used a survey method to learn about the note-taking behavior of post-secondary students revealed that almost all of the students reported taking notes in class (96%) and most students reported taking notes longhand in a notebook (86%). Additionally, 88% of participants reported that taking notes was necessary for effective learning (Morehead et al., 2019). This line of research demonstrates that the note-taking process is crucial to good academic performance in post-secondary education and should be further assessed in real-life courses.

Two models, by Flower and Hayes (1980) and by Bereiter and Scardamalia (1982, 1987), underlined the importance of metacognitive processes in writing. They described skilled writing in adults as primarily a metacognitive act. Both models discern expertise in writing largely with reference to the application of metacognitive processes such as planning (goal setting, generating, and organizing content), translating (converting ideas into text), and revising (changes made in text produced so far) (McCutchen, 1995). Moreover, a revised model by Hayes (1996) took into consideration the important role of working memory (WM) in the writing process. A number of researchers have argued that individual differences in writing skill stem from differences in WM resources (Berninger and Swanson, 1994; Berninger et al., 1994; McCutchen, 1996). New perspectives expand the metacognitive models, and argue that metacognitive ability is important but not sufficient for the development of writing expertise (Berninger et al., 1992, 1994; Ransdell and Levy, 1996; McCutchen, 2000). These extended views shed light on the importance of “efficient” or “fluent” execution of lower level processes in the execution of higher-level metacognitive processes. Writers must generate ideas fluently and write these ideas down quickly before they are forgotten. If writers master these skills, they will be able to use metacognitive processes to produce reader-based prose. Results from correlational and experimental studies with adults have systematically indicated that handwriting speed is significantly and positively related to the quality of essays. These findings have been expanded to note-taking, which is described as a vaguer, less cohesive, and more egocentric form of writing than essays (Brown et al., 1988; Olive and Kellogg, 2002; Connelly et al., 2005, 2006).

Despite the importance and value of note-taking, numerous studies have revealed that college students fail to record many important lecture points (e.g., Hartley and Cameron, 1967; Hartley and Marshall, 1974; Kiewra, 1984, 1985; Baker and Lombardi, 1985; Kiewra et al., 1987, 1988; Locke, 1977; O’Donnell and Dansereau, 1993). Furthermore, this failure to take complete notes has been shown to occur in more than one discipline. Students in psychology courses, for example, recorded less than 50% of lecture content (Baker and Lombardi, 1985). McDonald and Taylor (1980) showed that veterinary students also missed important ideas in lectures. It appears, therefore, that difficulties in producing comprehensive notes are common to a large number of students in more than one discipline and may impact academic performance.

### Note-Taking and Cognitive Skills

Note-taking has been shown to be dependent on a number of cognitive skills, including fluency transcription (hand-writing speed and spelling) and WM, among others (Kiewra et al., 1987; Kobayashi, 2005; Piolat et al., 2005; Peverly, 2006; Peverly et al., 2007). Relationships between the cognitive componence of note-taking have been investigated in a number of studies.

Working memory, or the ability to temporarily hold and manipulate limited amounts of information (Baddeley, 1986, 2007), appears to be an important component of note-taking. During note-taking, a load is imposed on WM, as visually and aurally acquired information must be held and organized (Bui and Myerson, 2014). The act of writing down this information, or encoding it in print, is believed to support recall (Kobayashi, 2005) in addition to providing an external memory store for later rehearsal (Eskritt et al., 2001). While this type of multi-tasking is clearly an important aspect of note-taking during lectures, findings on the contribution of WM to note-taking have been inconsistent. Some studies have found a significant positive relationship between verbal WM and note-taking quality (Kiewra et al., 1987; Kiewra and Benton, 1988; McIntyre, 1992), while others have found no relationship (Cohn et al., 1995; Hadwin et al., 1999; Peverly et al., 2007). These conflicting outcomes may be due to differences in the measures used to assess WM. Kiewra et al. (1987), Kiewra and Benton (1988) and McIntyre (1992) gave participants a set of six scrambled sentences and asked them to rearrange the words to make a sentence, or to make a coherent paragraph by arranging randomly ordered sentences. The materials were always in full view. These tasks differ from those used by researchers who reported significant correlation between WM and note-taking, based on the span test, a common measure of WM.

Handwriting speed, defined as the rate of written word production (Ransdell and Levy, 1996; Ransdell et al., 2002), is another cognitive ability that has been found to be related to note-taking skills (Peverly et al., 2007, 2013, 2014). Results from correlational and experimental studies of adults have systematically indicated that handwriting speed is significantly and positively related to the quality of essays. These findings have been extended to address note-taking, which is described as a vaguer, less cohesive, and more egocentric form of writing than essays (Brown et al., 1988; Connelly et al., 2005, 2006; Olive and Kellogg, 2002). A growing body of research suggests that handwriting speed is a significant predictor of the quality and completeness of notes (Peverly, 2006; Peverly et al., 2007, 2014; Peverly and Sumowski, 2012; Kodaira, 2017; Manzi et al., 2017) and that handwriting speed is positively correlated with note quality (Manzi et al., 2017). However, one study comparing post-secondary students with and without ADHD showed that the latter obtained lower scores than the former on tasks of written recall and handwriting speed, but did not differ from them in note quality (Vekaria and Peverly, 2018). Another study by Reddington et al. (2015), examined whether gender was related to note-taking ability in a large sample of undergraduate students (similar number of males and females). Results showed that females recorded significantly more information in notes and had better written recall than did males, and also performed significantly better on measures of handwriting speed, WM, language comprehension, and conscientiousness (a motivation measure).

### Language, Literacy, and Note-Taking

Despite developments in the field, a limited number of studies have examined the effects of language and literacy skills on note-taking ability among post-secondary students. Vocabulary is an important candidate in this context, given its critical role in post-secondary education, particularly while students listen to and attempt to document lectures. Vocabulary knowledge is considered to have two significant dimensions: breadth (the size of vocabulary or the number of words of which one has at least some superficial knowledge) and depth (how well one knows the words) (Read, 1989; Wesche and Paribakht, 1996; Qian, 1998, 1999). One study showed that language and broader academic achievements were positively influenced by vocabulary knowledge (Beck et al., 2002). Furthermore, clear links have been revealed between vocabulary and fluency of reading, comprehension, and achievement (Ehri and Rosenthal, 2007). Despite the value of vocabulary during science lectures (Flowerdew, 1992), there is little evidence of the role played by vocabulary in note-taking and lecture comprehension among students. Pioneering work examining the role of vocabulary in note-taking showed that middle school students with high achievements recorded 71% of 19 key vocabulary words from a lecture on average, while students with average achievements recorded 46%, and students with learning disabilities recorded 28% of in their notes. Moreover, of the variables examined in the study, vocabulary had the highest correlation with test scores (Boyle and Forchelli, 2014).

Another skill that is likely to play a role in note-taking is reading fluency, an important component of literacy. Fluency makes it possible for readers to focus attention on content while reading, rather than on the decoding of each individual word, such that automatic reading is directly related to reading comprehension (LaBerge and Samuels, 1974). Reading ability is considered a highly important skill in post-secondary education, during which information is often conveyed in lectures with both *oral* and *visual* channels. Namely, spoken lectures are frequently accompanied by relevant written material, such as key words and sentences, presented on PowerPoint slides (Microsoft, Inc.). Fajardo (1996) views note-taking as a complex activity that combines reading and listening with selecting, summarizing, and writing. Therefore, the ability to read is very important while taking notes. However, no studies have been conducted to measure the relationship between reading fluency and lecture note-taking.

Taking notes during a lecture is a very complex and cognitively demanding procedure (Piolat et al., 2005). Limited capacity processing has been determined to be a prevailing paradigm for the development of academic skills. Recent views of capacity limited cognitive processing (Marois and Ivanoff, 2005) and academic skills performance theories, which relate to reading (Hulme and Snowling, 2011), writing (McCutchen, 2000; Berninger, 2012), and mathematics (Geary, 2011), strongly suggest that the skill requires parallel operation of a hierarchy of domain-specific and higher order cognitive skills while WM capacity is limited. In order to use the limited capacity of WM, the basic skills of the specific domain must be sufficiently automatic and fluent, allowing application of the higher level cognitive skills needed to produce successful academic outcomes. Once lower-level skills related to note-taking are sufficiently fluent or automatic, for example WM, hand writing speed, and language based skills such as vocabulary, performing a higher order skill such as note-taking is possible.

### Note-Taking Among Second Language Learners

Given its relationships with various language skills, it stands to reason that note-taking is affected by bilingualism. With immigration on the rise, bilingualism has become an integral part of the educational reality for children in many parts of the world. Several studies have aimed to shed light on note-taking practices in academic contexts among second language (L2) students. This limited body of research has inferred that note-taking can facilitate the academic performance of L2 students, by enhancing their listening comprehension (Carrell, 2007; Hayati and Jalilifar, 2009; Song, 2011; Aminifard and Aminifard, 2012).

### L2 and Note Quality

Previous studies have examined the quality and features of notes taken by college students who spoke English as a second language. Fahmy and Bilton (1990) studied students learning English as their second language at Oman’s first university. Results indicated that 25% of L2 students took notes that were disorganized and had a poor layout. Another study (Clerehan, 1995) explored the differences between native (first language, L1) English learners and L2 non-native learners (international business students at Monash University (and revealed a large difference between the two groups in the hierarchical structure of the content they recorded. L1 students consistently recorded 99–100% of the principal elements, while L2 students omitted 19% of major headings, 34% of sub-headings, and 40% of legal cases, on average. Furthermore, Chaudron et al. (1988) showed a significant difference in the number of words recorded in the notes of English as L1 and English as L2 students, with the former recording 442 words on average and the latter 232 words. The number of words and ideas recorded in notes is positively correlated with higher achievements among both L1 and L2 students (Kiewra, 1987).

Research on the differences between L1 and L2 students in cognitive, literacy, and language skills, as they pertain to note-taking, is much more limited. A study on the relationship between WM capacities in L1 and L2 showed that the two shared substantial amounts of variance and that the significant relationship between them was not language-specific (Osaka and Osaka, 1992; Osaka et al., 1993; Juffs, 2005). However, a different pattern was obtained when differences between L1 and L2 WM capacity were assessed in relation to note-taking. One study by Piolat et al. (2005) examined the cognitive effort invested in note-taking while listening to passages in English. The results showed that note-taking was faster (as measured by reaction times) in the participants’ L1 (French) than it was in their L2 (English). Piolat (2005) argued that this result could be explained by the greater cognitive effort required for taking notes while listening to L2 passages, as compared to L1 passages, due to additional WM demands in L2. According to this explanation, L1 students would be expected to perform better than L2 students on the cognitive tasks underlying note-taking.

Limited vocabulary knowledge, specifically discipline-specific academic vocabulary, decreases the reading comprehension abilities of L2 students. A number of studies have established that vocabulary size is much more limited for L2 students, even in academia after many years of formal exposure to the L1 language (Gollan et al., 2002; Portocarrero et al., 2007; Bealle et al., 2008). However, to our knowledge, no study has examined the role of vocabulary in note-taking among L2 students. Alongside vocabulary knowledge, oral reading fluency (ORF) is another skill that poses challenges for L2 students in post-secondary education. Any reading activity assigned in a post-secondary classroom for an academic purpose is defined as an academic literacy task (Carrell and Carson, 1997). However, to our knowledge, no studies have examined ORF, L2, and note-taking.

Note-taking in L2 appears to be a uniquely challenging task. Besides the difficulties facing L1 students, L2 students also face specific difficulties in organizing and designing notes, writing full and complete information, recalling lecture concepts and details, and writing them down simultaneously with an appropriate level of language proficiency. Furthermore, L2 students have a limited vocabulary compared to L1 students, and spend more cognitive effort than do L1 students during the process of taking notes, which affects their note quality and test achievements.

### The Case of Israel

In Israel, most of the native Arabic-speaking minority has limited formal exposure to Hebrew (L2 in this case), and especially to academic and written language, before and during primary and secondary school. Arab sector schools generally start teaching Hebrew in the second grade, and provide a limited number of Hebrew teaching hours in primary schools (3 h per week) and middle and high school (2–5 h per week) (Israel Ministry of Education, 2010). Thus, students who speak Arabic as a first language (Hebrew as L2) are expected to face difficulties in reaching a high level of Hebrew proficiency, and to meet many challenges when required to study in Hebrew upon reaching post-secondary education.

Over the past four decades, the number of Arabic-speaking students in Israel’s higher education system has increased dramatically. According to the Central Bureau of Statistics (2017), in the 2015–2016 school years, 7,665 Arabic-speaking students (10.2% of all students) received academic degrees from institutions of higher education in Israel. Among recipients of undergraduate and master’s degrees, there was an increase in the percentage of Arabic speakers compared to 2014/15 (from 10.2 to 11.0% and 9.2 to 9.3%), respectively. Among those receiving a Ph.D. degree, there was a decline in the number of Arabic-speaking graduates (from 4.4 to 4.1%). In light of this trend, it is clearly important to investigate the specific challenges faced by L1 Arabic-speaking students in developing and implementing academic skills, among them the substantial task of note-taking.

To summarize, overall, the literature suggests that note-taking is a complex process for many students, which requires various cognitive, language, and literacy skills. Pioneer studies have indicated that cognitive skills including WM and handwriting speed underlie this process. However, in-depth research is required on the various processes and skills affecting note-taking abilities within different populations. While there is evidence suggesting that vocabulary and word reading fluency affect note-taking, little work has addressed the relationships between these variables directly.

### The Current Study

The current study aimed to examine the relationships between cognitive, language, and literacy skills and note-taking ability among post-secondary students with Hebrew as their first versus their second language. This research extended the work of Peverly et al. (2007, 2013, 2014) by measuring additional cognitive, literacy, and language measures, namely word reading fluency, and vocabulary size.

The specific research questions addressed were as follows:

(1) Which of the cognitive, language, and literacy skills are associated with note quality among all students? Are there differences in the correlations between note quality and other variables? Based on a review of the relevant literature, and given the importance of the variables in note-taking, the hypothesis was that all cognitive, language, and literacy skills would be associated with note quality.
(2) Are there differences in note quality between L1 and L2 students?
  We hypothesized that note quality would be higher among L1 students than among L2 students.
(3) Are there differences in the various cognitive (hand writing speed, WM), literacy (fluency of word reading), and language (vocabulary) skills between L1 and L2 students?

We predicted that a significant difference would be found between the two groups in literacy and language skills (language based skills). We further predicted that there would be a significant difference between groups in all cognitive skills except WM, with L1 students outperforming L2 students.

## Materials and Methods

### Participants

Sixty-three first-year university undergraduate students participated in this study. As evident in Table 1, the participants included 28 L1 students (94.3% female) and 35 L2 students (92.9% female). Of the participants, 79.4% were 20–25 years old, 19% were 26–30 years old, and 1.6% were older than 30. Participants were recruited from the Faculties of Education (89%) and Social Sciences at the University of Haifa, and had not been diagnosed with any learning disability or with attention deficit-hyperactivity disorder. In return for their participation in the study, students received extra course credit. All students were capable of taking notes manually. The study received ethical approval. Before beginning the experiment, all participants received an explanation about the study and were informed that they would be free to terminate participation at any point in the experiment, and that their identity would remain anonymous.

**Table 1:** Descriptive statistics for first (L1) and second (L2) language participants.

		L1 students	L2 students	All students
**Number**		28	35	63
**Age**	**20–25**	94.3%	60.7%	79.4%
	**26–30**	2.9%	39.3%	19%
	**31+**	2.9%	–	1.6%
**Gender**	**Female**	94.3%	92.9%	93.7%
	**Male**	5.7%	7.1%	6.3%


### Procedure

Four university lecturers were contacted, of which two agreed to participate in the study. Students received credits for participation in the experiment, with the agreement of the lecturers. After receiving approval for participation in the study, the students were invited to take part in two sessions. The first was conducted at the end of each lecture, in coordination with the lecturers. This session was administered collectively: all participants were asked to read a short explanation about the nature and procedure of the study (5 min), and then to view a videotaped lecture (with no Power Point presentation) on a large color projector in the classroom, during which they were asked to take notes. The lecture topic was selected from an online sociology course dealing with bureaucratic organizations. This topic was chosen because it is usually presented during the second year of academic studies and is relatively unfamiliar to first-year students from the Faculty of Education (the study population). To ensure anonymization the notebooks were numbered and each student received a sticker with a number corresponding to the notebook s/he used. Participants were asked to keep the numbered sticker in order to identify them in the second part of the study. Before beginning observation, the experimenter explained to the participants that they would watch a real videotaped lecture and asked them to listen carefully and to take notes with a pencil and paper. They were also asked to behave as if they were in a real classroom, as though they would be using the notes to study for exams. The lecture lasted 10 min. All notes were collected by the experimenter. Following the lecture, participants were asked to fill out a brief demographic questionnaire (5 min). The second part of the study was conducted individually with the experimenter over the next 5 weeks. In this session, participants performed cognitive tasks including handwriting speed, and WM, followed by a literacy task (word reading fluency) and a vocabulary task. The individual sessions were conducted in a quiet room at the university and took approximately 25 min.

### Measurements

#### Background and Demographic Questionnaire

The questionnaire (see Supplementary Appendix A) included information on: gender, age, diagnoses, educational background, language background, and ethnicity. It was administered collectively to all students in the classroom.

#### Note Quality

The videotaped lecture on Bureaucratic Organizations was chosen from among the online courses available at the University. The criteria for choosing the lecture were: (1) clear tone and appropriate speed of speech, (2) clear structure, and (3) a presumably unfamiliar topic that would be studied at a later stage, usually in another department. The lecture was presented at an average rate of 110 words per minute on a large color projector in the classroom. The scoring method was taken from Brobst (1996). The lecture included 15 content areas. For each content area, notes were given between 0 and 3 points, in accordance with the extent to which they addressed the main topic in a clear way. The scoring grid is presented in Supplementary Appendix B. For example, one item addressed the definition of the term bureaucracy. To receive 3 points, participants had to present a full explanation (e.g., “The concept of bureaucracy was introduced in 1745. This term is a combination of two Greek words: “bureau” which means a desk or office, and “cracy,” which means rule; thus the meaning of the word is official rule.”). Students received 2 points if the topic was mentioned with a partial explanation (e.g., “The concept of bureaucracy was introduced in 1745. This term is a combination of two Greek words: “bureau,” which means a desk or office, and “cracy,” which means rule.”) If the topic was mentioned but no elaboration was provided, the participant received 1 point (e.g., “The concept of bureaucracy was introduced in 1745,”) and no points were given for incorrect or missing information. All other examples of “bureaucracy’s model features” by Weber were rated on a scale of 0 to 2 points. Participants received 2 points for mentioning one feature of the bureaucracy model (e.g., the division of labor in the organization) and providing further information about it (e.g., work is divided between the various employees, and becomes an official duty of each position holder). One point was given if an example was provided (e.g., the division of labor in the organization) without elaboration, and no points were given for incorrect or missing information. Note quality could range from 0 to 48 and the maximum score in the current sample was 47. Thus, low versus high quality was determined by the number of main ideas that were included, using proper syntax, in the notes. Two raters analyzed a sample of 15 notes that were randomly chosen. The raters were not exposed to the language status of the participants. Upon completion of the analyses, the raters compared the scores and, according to the protocol, if there were differences of more than five points, a discussion was held and the disagreements were settled by consensus. Inter-rater agreement, assessed by SPSS intra-class correlation across 15 randomly chosen protocols and between two independent scorers, was 0.93.

### Cognitive Measures

#### Handwriting Speed

This task was adapted from Peverly et al. (2013), who adapted it from an original task developed by Berninger et al. (1991). In the original study, children were asked to write as many letters of the alphabet (A to Z) as they could in 30 s. In the current study, similar to Peverly et al. (2013), participants were asked to write as many letters of the Hebrew (the target instruction language) alphabet as possible, starting with the first letter “
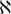
” and ending with the last letter “
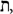
” on a lined sheet of paper in 45 s. Each recognizable letter received one point, and the points were summed to calculate a total score for each participant. The maximum score was 90.

#### Working Memory: Letter Number Sequencing Test

To assess verbal WM, a Hebrew version of the Letter-Number Sequencing test from the Wechsler Memory Scale (WAIS-III, Wechsler, 1997) was used. During the test, a combination of numbers and Hebrew letters were read by the experimenter from right to left, at a rate of approximately one item per second. Participants were required to listen to the experimenter and then to repeat back the numbers in ascending order, followed by the letters in alphabetical order.

The test ended when the participant failed to retrieve any of a series of a certain capacity (for example, when s/he could not correctly repeat any of a series of six items). A correct retrieval received 1 point and an incorrect retrieval received 0 points. The maximum score was 21.

#### Literacy and Language Measures

##### Word reading fluency

The Test of Word Reading Efficiency (TOWRE; Schiff et al., 2006; adapted from Torgesen et al., 1999) was used. In the test, 78 Hebrew real words of increasing difficulty were arranged in four columns. Participants were required to read aloud as many words as possible within 45 s. This is a timed word recognition test that measures word reading fluency. Scores ranged from 0 to 78, reflecting the number of accurate words the participant read within the time limit.

##### Vocabulary

Hebrew vocabulary was measured using an adaption of Peabody’s Picture Vocabulary Test (PPVT). The Hebrew version was standardized by Solberg and Nevo (1979) and contains 110 items ranked according to level of difficulty. Each item is comprised of four black-and-white pictures. The examiner read a word and the participant had to point to the picture corresponding to that word. Correct answers received 1 point and incorrect answers received 0 points. The test ended when the participant answered six out of eight consecutive items incorrectly. The split-half reliability of the Hebrew version is 0.90. Results are reported in terms of the number of correct responses. The minimum obtainable score on the PPVT task is 43 and the maximum score is 108.

## Results

SPSS version 21 was used to analysis the data. To address the first question, Pearson correlations were used to measure the relationship between note quality and the cognitive, language, and literacy variables. To address the second question regarding differences in the quality of notes between the two groups, a *t*-test was used, with group (L1 and L2 students) as the independent variable and quality of notes as the dependent variable. A MANOVA was also used to address the third question, on the effects of first language (L1 and L2; independent variable) on cognitive (WM, handwriting speed, rapid automatic naming task), literacy (word reading fluency), and language (vocabulary) variables (dependent variables). A follow-up ANOVAs with a Bonferroni correction was conducted.

The first goal of the study was to assess the relationship between note quality and the literacy, language, and cognitive variables. Pearson correlations were calculated to assess the relationships between note quality and each of the variables. Correlations for the full sample are presented in Table 2.

**Table 2:** Pearson correlations between note-quality, cognitive, literacy, and language variables for the total study sample (*N* = 63).

Variable	1	2	3	4	5	6
(1) Quality of notes	–					
(2) TOWRE Word Reading	0.60^∗∗^	–				
(3) Vocabulary	0.60^∗∗^	0.75^∗∗^	–			
(4) WM	0.34^∗∗^	0.32^∗^	0.42^∗∗^	–		
(5) HSpeed	0.37^∗∗^	0.53^∗∗^	0.41^∗∗^	0.07	–


*TOWRE, test of word reading efficiency (word reading fluency measure); vocabulary, size of vocabulary; WM, working memory; HSpeed, handwriting speed. *^∗^p* < 0.05, *^∗∗^p* < 0.01.*

As shown in Table 2, note quality was significantly correlated with all the other variables in the entire sample *(N* = 63). Note quality showed strong positive correlations with word reading fluency, *r*(61) = 0.60, *p* < 0.01, and vocabulary, *r*(61) = 0.60, *p* < 0.01). Moderate positive correlations were found between note quality and WM*, r*(61) = 0.34, *p* < 0.01, and between note quality and handwriting speed, *r*(61) = 0.37, *p* < 0.01. Thus, higher note quality scores were correlated with better word reading fluency, a larger vocabulary, better WM, and faster handwriting.

The second goal of the study was to examine whether there were differences in note quality between L1 and L2 students. To answer this question, a *t*-test was conducted. The dependent variable was Note Quality, based on the total number of main ideas written in proper syntax in the notes. There was a significant difference in note quality between the two groups, *t*(61) = -5.15, *p* < 0.05, with L1 students (*M* = 28.18, *SD* = 5.60) producing notes of higher quality than those of L2 students (*M* = 18.74*, SD* = 8.86).

The third goal was to examine the differences and effects of the various cognitive, literacy, and language (vocabulary) skills between L1 and L2 students. Table 3 presents the means and standard deviations of the literacy, language, and cognitive variables, which were compared between the two groups using MANOVAs. Overall, as expected, the results demonstrate a significant difference in first language status (L1 vs. L2) based on students’ literacy, language, and cognitive skills [Wilk’s Λ = 0.279*, F*(4,58) = 37.53, *p* < 0.001, η^2;^*p* = 0.721]. Follow-up ANOVAs with a Bonferroni correction (*p* < 0.01) indicated significant differences between groups on word reading fluency [*F*(1,61) = 75.399, *p* < 0.001; η^2;^p = 0.55], vocabulary scores [*F*(1,61) = 98.88, *p* < 0.001; η^2;^*p* = 0.618], and handwriting speed [*F*(1,61) = 30.67, *p* < 0.001, η^2;^*p* = 0.34]. There were no differences between the groups on the WM task, *F*(1,61) = 3.96, *p* = 0.51). L1 students read words more fluently, had higher vocabulary scores, and wrote faster than L2 students, while no significant differences were found in the WM task.

**Table 3:** Means and standard deviations for literacy, language, and cognitive variables among Hebrew as a first language (L1) versus Hebrew as second language (L2) students.

	Hebrew as first language (*n* = 28)		Hebrew as second language (*n* = 35)	
		
Variable	*SD*	*M*	*SD*	*M*
TOWRE Word Reading	64.29	12.41^*^	40.54	9.29
Vocabulary	96.32	6.64^*^	67.77	13.96
WM	13.39	2.48	12.31	1.81
HSpeed	68.79	15.13^*^	46.80	16.02


*TOWRE, test of word reading efficiency (word reading fluency measure); Vocabulary, size of vocabulary; WM, working memory; HSpeed, handwriting speed. *^∗^p <* 0.001.*

This finding is consistent with similar findings published in a number of limited studies that examined the quality of L2 note taking. These studies found that L1 students outperformed L2 students in recording principal elements in their lecture notes.

## Discussion

Note-taking while listening to lectures is considered a complicated skill with vast importance in post-secondary education (Dunkel and Davy, 1989), which requires students to utilize a number of capabilities. A high percentage of students fail to record important ideas while taking notes, which in turn affects their academic achievements. Previous studies conducted by Peverly et al. (2007, 2013) demonstrate that two cognitive variables are related to note-taking: WM and handwriting speed. The current study aimed to extend the work of Peverly et al. (2007, 2013, 2014), and to expand our understanding of the different variables underlying note-taking skills. Therefore, alongside the cognitive variables mentioned above, additional literacy (word reading fluency of reading words) and language (vocabulary) variables were examined. Furthermore, the study aimed to shed light on differences between note-taking skills in L1 and L2 by examining Hebrew-speaking L1 and Arabic-speaking L2 students at a predominantly Hebrew-speaking university.

### Note-Taking and Cognitive, Literacy, and Language Skills

The first goal of the study was to examine which of the cognitive, language, and literacy skills are associated with note quality among all students. Overall, the current study confirmed that cognitive, language, and literacy variables were correlated with note quality in the entire sample. Specifically, word reading fluency and vocabulary size had strong positive associations with note quality. Moderate positive correlations were found between note quality and WM and between note quality and handwriting speed. Existing research on the relationships between vocabulary, word reading fluency, and note quality is very limited. A previous study by Boyle and Forchelli (2014) addressed the correlation between test scores and vocabulary recorded in notes, but not the correlations between vocabulary and note quality. The current study was the first, to our knowledge, to examine correlations between vocabulary and note quality among post-secondary students. The current results shed light on the importance of increasing vocabulary size to enhance note quality among all post-secondary learners. Word reading fluency has also become a critical skill in post-secondary education, particularly because of the widespread use of PowerPoint slides and the high prevalence of lectures with both *oral* and *visual* channels (Palkovitz and Lore, 1980; Doyle, 2011). A previous study indicated a relationship between note-taking and reading from slides presented during a spoken lecture, specifically showing that the act of reading enables note-takers to record and retain more relevant information (Haynes et al., 2015). The results of the study support this result confirming the relationships between word reading fluency and note taking skills.

With regard to WM, the results of the current study were similar to those reported by Peverly et al. (2007) and other previous studies that examined the correlations between WM and note-taking (Cohn et al., 1995; Hadwin et al., 1999) but contradicted others (Kiewra et al., 1987; Kiewra and Benton, 1988; McIntyre, 1992). The conflicting outcomes may be due to differences in the tools used to assess WM. In the current study, WM assessment was not limited in time, which might lessen the strain on the resources required to maintain information. The studies that showed no relationship between WM and note-taking skills used a span test to assess WM. This test required participants to process and store information in a different way than the task used by Kiewra and Benton (1988), Kiewra et al. (1987) and by McIntyre (1992), who gave participants a set of six scrambled sentences and asked them to rearrange the words to make a sentence, or to form a coherent paragraph by arranging randomly ordered sentences. With regard to handwriting speed, the current results support the results of Peverly et al. (2014) and another results by Manzi et al. (2017), which reported correlations between note quality and handwriting speed, showing that higher handwriting speed is associated with better note quality. That is, transcription fluency is important to recording the ideas presented in the lecture.

Overall, the results demonstrate the multi-component approach to note-taking skill by examining language, literacy and cognitive skills. Although many studies presented theoretically that note-taking is a complicated skill that requies many skills, the current study took the first step in addressing this claim empirically.

### Quality of Notes

The second goal of the study was to investigate if there are differences in note quality between L1 and L2 students. The results demonstrated differences between L1 and L2 with regards to the quality of notes taken during a videotaped lecture. The results demonstrated that L1 students produced higher quality notes than did L2 students. In other words, they produced a larger number of main ideas written in proper syntax than L2. This finding is consistent with similar findings published in a limited number of studies that examined the quality of L2 note- taking. These studies found that L1 students outperformed L2 students in recording principal elements in their lecture notes (Fahmy and Bilton, 1990; Clerehan, 1995), and reflects the unique difficulties faced by L2 students during note-taking.

Note quality was found to be better among L1 students than among L2 students in the present study and as this is one of the crucial skills at the post-secondary education, support services should take into consideration.

### L1 and L2 Differences

The third goal of the study was to examine if there are differences in the various cognitive (hand writing speed, WM), literacy (fluency of word reading), and language (vocabulary) skills between L1 and L2 students. Differences between L1 and L2 students were found with respect to various language, literacy, and cognitive variables. L1 students performed significantly better than L2 students on the vocabulary task. This finding is in accordance with a previous study indicating a large difference between the vocabulary sizes of L1 and L2 students, which worked to the benefit of L1 students during their first year in academia (Rosselli et al., 2000; Gollan et al., 2002; Portocarrero et al., 2007; Bealle et al., 2008).

Confirming our hypothesis, the results of the current study also demonstrated that L1 students performed better than L2 students in word reading fluency. The current findings indicate the difficulties facing L2 students with fluent reading of words. Therefore, the insufficient exposure to the Hebrew language may directly affect L2 reading performance.

With regard to the additional cognitive skills examined in the study, there was a difference between the two groups in handwriting speed. This result is similar to the results of Barbier and Piolat (2005), who showed that note-taking was faster (as measured by reaction times) in L1 (French in this case) than in L2 (English) when listening to passages in L2. Another study (Faraco et al., 2002) also showed a difference between non-native students (German and Spanish freshmen) and native students (French freshmen) in word transcription fluency, in favor of the native students.

The current results showed that there were no differences between the two groups in WM, in accordance with previous work showing that L1 and L2 WM capacities share substantial amounts of variance and that the relationship between the two was not language-specific (Osaka and Osaka, 1992; Osaka et al., 1993; Juffs, 2005). These findings support the possibility that L1 and L2 students share similar WM resources.

## Conclusion

The results of current study extend our understanding of note-taking among both post-secondary students and second language students. First, the results demonstrated the contribution of the language, cognitive, and literacy variables to note quality among post-secondary students in general. The study illuminated important variables underlying note-taking skills, namely: vocabulary, word reading fluency, WM, and handwriting speed.

Second, the study revealed that L1 students performed significant better than L2 students on note quality. Furthermore, the study examined L1 and L2 students with respect to literacy, language, and cognitive skills as well as note-taking. L2 students had lower scores than did L1 students in vocabulary size, word reading fluency and handwriting speed, though the groups performed similarly, on WM tasks.

These findings contribute to the literature examining different variables underlying note-taking during university-level lectures by looking at note-taking from a multi-component percepctive. Furthermore, they shed light on the difficulties specifically facing L2 post-secondary students when taking lecture notes, and can guide support centers at the universities and colleges in the development of appropriate intervention programs. It is possible that L2 students fail to record the main ideas discussed in lectures due to an inability to follow the rate of speech or to insufficient understanding of academic terms in their second language. Given the known advantages of note-taking, it is clearly important to enhance this skill as a means of improving learning experiences and outcomes among L2 students. Vocabulary is of major importance in academic life, such that insufficient knowledge in this area might increase the difficulties facing L2 students and affect the quality of their notes and their educational achievements. Therefore, there is a need to develop academic intervention programs aimed at enhancing academic vocabulary specifically among L2 students in post-secondary education.

This study has both theoretical and practical implications. The results broaden existing knowledge regarding the factors affecting the performance of L1 and L2 students in their first year in post-secondary education. As such, the findings may contribute to the development of optimal teaching methods, and to the establishment of support programs aimed at helping students to improve the quality of their notes and thereby achieve better academic performance. More specifically, the findings suggest that such programs should focus on improving reading fluency. The results also suggest courses aimed at helping L2 students enhance handwriting speed and note-taking skills or alternatively for example by using a tape recorder or notes taken by a peer.

In addition, these results pave the way for future studies examining: (1) additional variables underlying note-taking skills; (2) the quality of notes in at-risk groups such as students with learning disabilities; (3) the effects of lecture medium (regular versus online) on the quality of notes; and (4) support programs for note-taking skills of L2 students.

### Limitations and Future Studies

The current study has several limitations. First, there was a greater number of female participants than male participants in both the L1 and L2 samples. In addition, the current sample included students in their first year of academic studies. Future studies should also take into consideration the changes that occur in students over the course of their academic studies, from changes in vocabulary size and literacy abilities to changes in the other cognitive abilities underlying note-taking skill. It may be that the nature of the videotaped lecture affected these results, as students could not ask the lecturer to speak more slowly or repeat certain words, or ask clarification questions. Another limitation is related to the assumption of low familiarity with the lecture topics. Familiarity was not directly assessed in the current study, but should be rated in future work as it could potentially influence the quality of note-taking.

The current study examined two languages that use different alphabetical systems. This may have impacted the performance of the participants (e.g., handwriting speed, reading fluency) and thus impeded direct generalization of the results to cases in which L2 shares the same alphabet as L1.

Future studies should attempt to replicate our results and include more variables that are likely to be relevant to note-taking, in order to build a comprehensive model of this important skill. Additional directions worth pursuing include examination of note organization and assessment of note-taking abilities among L1 and L2 students when different teaching methods are used, such as lectures with slideshows and outlines.

## Ethics Statement

This study was approved by the Research Ethics Committee of the faculty of Education at the University of Haifa.

## Author Contributions

MA-Z and OL contributed to the conceptual framework, statistical analyses, writing the manuscript and agreed to be accountable for the content of the work. MA-Z made contributions in task development, data collection and statistical analysis.

## Conflict of Interest Statement

The authors declare that the research was conducted in the absence of any commercial or financial relationships that could be construed as a potential conflict of interest.
