# Supplementary material for: Note-Taking Skill Among Bilingual Students in Academia: Literacy, Language and Cognitive Examination
Source: Front Psychol. 2019 May 14;10:870. doi: 10.3389/fpsyg.2019.00870 (PMC6527783; doi:10.3389/fpsyg.2019.00870)
Supplement: Supplementary file 1 [file Data_Sheet_1.docx]

**Appendixes**

**Appendix A:**

Demographic questionnaire

Demographic questionnaire

Student number: _____

*Personal Information*

a. Gender: 🞏 male 🞏 female

b. Age group: 🞏 20-25 🞏 26-30 🞏 over 30 🞏 else__________

c. Department of study: ______________________________

d. Present academic year: 🞏 First 🞏 Second

e. Years of education: ______________________

f. Post-secondary institution: _________________

g. Have you been diagnosed with learning disabilities or attention deficit-hyperactivity disorder? Yes \ No.

h. Mother tongue: 🞏 Hebrew 🞏 Arabic 🞏 Other

i. Language proficiency level: 🞏 High 🞏 Medium 🞏 Low

j. Dominant hand: 🞏 Right 🞏 Left

**Appendix B:**

Note quality scoring

***Note quality scoring***

| *Organizations as bureaucratic mechanisms* | | | |
| --- | --- | --- | --- |
| Scoring  (3 points for each content area) | | | |
| 1 | 1 | 1 | Contents areas |
| The meaning of the term is official rule. | This term connects two Greek words; "bureau" which means a desk or office, and a "cracy" which means rule. | The concept of bureaucracy was introduced in 1745. | 1. Definition of the concept of bureaucracy. |
| Theorists called it bureaumania (due to the great power of those officials). | Theorists who used this term viewed the phenomenon as a social illness. | The initial use of the term "bureaucracy" was to describe the power created by government officials. | 1. Term usage. |
| The use of this term by both authors stems from and relates to the field of sociology; it deals with official organizations that have become inefficient and cumbersome over time (in this context, there has been a tendency to use bureaucratic terms). | Kafka is another author who frequently used this term in his book "The Law."  His writings deal with the situation of modern man facing powerful bureaucratic systems. | Balzac is a French author who used the term bureaucracy in his books and provided criticism of the nature of the organizations. | 1. Theorists who described the power created by government officials. |
| From the various organizational forms, he thought that bureaucracy as a formal form in organizations is the most effective. | He studied governmental, commercial, church and party organizations intensively. | Max Weber as a researcher of organizational forms often used the term bureaucracy. He was a German sociologist in the 19th century. | 1. Max Weber as a researcher of organizational forms. |
| He also worked in small and informal organizations (held in any organization). | These relationships are defined within the regulations and regulated within the organization. | He gave a central place to the formal and advanced relations within the organization. | 1. Weber's practice in "formal bureaucratic and informal organizations." |
| Effective organization has a social regime based on the principle of law and order. | This model emphasizes the appropriate and exclusive ways (from his point of view) to establish an effective organization. | Max Weber developed his own model and called it the "ideal model" of the organization. | 1. Weber's ideal model. |
| But rather a certain necessity that ensures a regime and order in society and leads to stability in organizations. | The rules and procedures are not the result of arbitrary coercion. | The principle of law and order is based on the consent of the organization to comply with rules and procedures. | 1. What is the principle of law and order? |
| *Weber's ideal model properties (two points per property)* | | | |
| Scoring  (Two points for mentioning the property and its definition) | | | |
| 1 | | 1 | |
| The work is divided between all of the workers. These jobs become the official duties of every worker. | | 1. Labor division in the organization. | |
| The division of labor between workers must be clear and enables specialization among the workers, each in his or her field of expertise | | 1. A clear division of labor. | |
| The jobs are organized in an authoritative pyramidal structure in which each official and other workers in the organization are responsible for the decisions of those subordinate and superior to them. | | 1. A clear system of multilevel authority that leads to command and subordination. | |
| The authority of each worker is determined by a set of accurate rules and regulations, in which each worker knows, in advance, his responsibilities and how he must behave toward superiors and subordinates. | | 1. Defining the authorities of the workers. | |
| This separation should lead to impersonal relationships. Therefore, customers or subordinates must be treated with objective and impersonal orientation. | | 1. Complete separation between official activities and private life. | |
| The work in the organization is a life service. Through this work one can develop a professional career. Every hired worker must be assigned a position according to his qualifications and areas of specialization. Then they must be guided to fulfill roles, understanding that what they do is a service and commitment. | | 1. Service for lifetime. | |
| The employment must be based solely on the employees’ technical skills rather than on family-political and social relationships. These skills must be tested through standard measurements. | | 1. Workers employment in the organization. | |
| Promotion is based solely on professional skills related to the position. | | 1. Promotion. | |
